# Telehealth and Outpatient Visits Among Individuals with Chronic Conditions by Socioeconomic Status in the First Year of the COVID-19 Pandemic: Observational Cohort Study

**DOI:** 10.1089/tmj.2022.0233

**Published:** 2023-07-04

**Authors:** Aliza S. Gordon, Yeunkyung Kim

**Affiliations:** Enterprise Health Services Research, Elevance Health, Inc., Indianapolis, Indiana, USA.; ^*^ Department of Healthcare Administration and Policy, University of Nevada, Las Vegas, NV.

**Keywords:** telehealth, telemedicine, COVID-19, socioeconomic status, outpatient utilization

## Abstract

**Objective:**

We investigated telehealth usage for individuals with chronic conditions by neighborhood-level socioeconomic status (SES) during the first year of the COVID-19 pandemic.

**Methods:**

We split the population of 2.3 million commercially insured adults in the United States with at least one chronic condition in claims into four quartiles of SES using address of residence. After balancing groups on baseline characteristics, we examined telehealth and total outpatient evaluation and management (E&M) visits from March 2020 to February 2021.

**Results:**

Quartile 4 (highest SES) had more telehealth visits per person (0.054–0.100 more visits over each 3-month period) and a higher percentage of visits that were telehealth (1.8–5.9 percentage points higher) than other quartiles. Quartile 4 had higher levels of total outpatient E&M use throughout the year. Differences in telehealth between Quartiles 1 and 3 were small.

**Conclusions:**

Commercially insured individuals in the highest SES quartile had higher use of telehealth and total E&M visits than other quartiles.

## Introduction

The onset of the COVID-19 pandemic in the United States brought a rapid increase in telehealth. However, there may be disparities with lower telehealth usage among more vulnerable populations; earlier studies indicate that racial/ethnic minorities,^1–3^ individuals without a college degree,^2^ individuals living in lower income neighborhoods,^1,4,5^ and individuals without private health insurance^2,6^ were less likely to use telehealth during the early months of the pandemic. Access to telehealth may be lower in neighborhoods with lower socioeconomic status (SES) because of reduced access to broadband internet, connected devices, and video-capable technologies.^3^ Access to high-quality telehealth may be further reduced in low SES neighborhoods because of what health care providers offer; one study demonstrated that providers in areas with a high social vulnerability index were less likely to adopt video telehealth and more likely to adopt audio-only telehealth.^7^

The evidence regarding telehealth use by SES during the COVID pandemic in the United States is mainly limited to the first few months of the pandemic (through June 2020 or earlier).^1,3–7^ It is possible that access to telehealth improved during the course of the pandemic for vulnerable populations, particularly if providers in their neighborhoods were able to offer more telehealth services over time. Furthermore, questions remain regarding how the adoption of telehealth by SES affected overall outpatient health care utilization. Our study therefore aimed to compare the usage of telehealth and total outpatient visits among individuals with chronic conditions by neighborhood-level SES during the first full year of the pandemic.

## Materials and Methods

### DATA AND STUDY POPULATION

Our data source was medical claims from the HealthCore Integrated Research Environment (HIRE^SM^), a repository of fully adjudicated medical and pharmacy claims data for members enrolled in health plans managed by a large insurer across the United States.

Our sample consisted of commercially insured members with a diagnosis of diabetes, chronic kidney disease, cardiovascular disease (congestive heart failure or atherosclerotic cardiovascular disease), asthma, chronic obstructive pulmonary disease (COPD), or a musculoskeletal condition (osteoarthritis or back pain) on at least 2 dates in claims between March 2019 and February 2020 (at baseline). We focused on these conditions because patients need regular management, and changes in routine care could increase the risk of exacerbation of the conditions over time. Furthermore, those with diabetes, kidney disease, cardiovascular disease, or respiratory conditions were at higher risk for poor outcomes after COVID-19 infection,^8^ so they may have been more concerned about contracting COVID-19, and therefore, more likely to skip or delay in-person care or use telehealth instead. Continuous enrollment in the health plan from March 2020 to February 2021 was required.

### EXPOSURE

We split the population into four quartiles of SES based on a composite score^9^ generated from seven variables from the 2018 American Community Survey, using address of residence at the census block group level.

### OUTCOMES

We examined claims for all-cause outpatient evaluation and management (E&M) visits (in-person and telehealth), and telehealth visits specifically, during four 3-month periods from March 2020 to February 2021. Telehealth visits were defined by either a Current Procedural Terminology (CPT) code specific to outpatient telehealth; Centers for Medicare & Medicaid Services Place of Service Code = “02” during an outpatient E&M visit; or procedure modifier code of “GT,” “GQ,” “G0,” or “95” during an outpatient E&M visit. We also identified a subset of telehealth visits that were audio only (CPT codes 98966-8, 99441-3), although it is likely that some providers used other telehealth codes for audio-only telehealth visits (i.e., this is likely an underestimate of audio-only visits). Telehealth visits were not subject to cost sharing for most of the study population during the study period; median patient payments were $0 and mean patient payments were $11.01 (see *Supplementary Appendix SA1* for further details).

### ANALYSIS

Inverse probability weighting (IPW) was used to balance the four groups on sex, age, U.S. Census Division, baseline Elixhauser comorbidity index (ECI) score (during March 2019 to February 2020), and chronic condition(s) of interest. This allowed for a direct comparison of patients with similar demographic and clinical characteristics except for SES. Weighted *t*-tests were then used to compare differences in telehealth and total outpatient E&M use by SES quartile. We then repeated the analysis above, including a new IPW analysis, using deciles of SES score rather than quartiles, to gain a more granular view of the relationship between SES and utilization. Analyses were conducted in SAS Enterprise Guide 7.1.

### ETHICS

This study was conducted using a limited dataset for analysis which was devoid of individual patient identifiers and complied with all relevant provisions of the Health Insurance Portability and Accountability Act (HIPAA) and the HIPAA Privacy Rule (45 CFR 164.514(e)). The study was exempt from Institutional Board Review.

## Results

### STUDY POPULATION

Our sample included 2,292,624 individuals, split into 4 SES quartiles, where 1 is the lowest SES. Unweighted baseline characteristics may be found in *Supplementary Appendix SA2*. After weighting, the sample was 51.2% women, had a mean age of 51.1 (standard deviation [SD] 34.0), and a mean ECI score of 2.66 (SD 4.56) (*Table 1*). About 53.0%, 30.9%, 16.8%, 14.8%, 6.0%, and 4.4% of the sample had a baseline diagnosis of a musculoskeletal condition, diabetes, cardiovascular disease, asthma, chronic kidney disease, and COPD, respectively. South Atlantic and Pacific were the most common Census Divisions, at 22.1% and 20.3%, respectively. Each weighted quartile was balanced on all non-SES–related baseline characteristics (standardized differences all <0.01). The average median household income by census block group was $45,535, $65,457, $84,809, and $128,930 in SES quartiles 1–4, respectively; the mean proportion of those who graduated from high school by census block group was 0.754, 0.875, 0.928, and 0.965 in SES quartiles 1–4, respectively.

**Table 1.** Baseline Characteristics of the Weighted Quartiles of Socioeconomic Status

SES QUARTILE	1	2	3	4	TOTAL	LARGEST STANDARDIZED DIFFERENCE^a^
*N*	573,156	573,052	573,253	573,163	2,292,624	
Weighted *n*	2,273,522	2,273,742	2,274,094	2,277,504	9,098,863	
Age, mean (SD)	51.04 (32.69)	51.11 (33.76)	51.10 (34.35)	51.13 (35.23)	51.09 (34.02)	0.003
Female, *n* (%)	1,162,602 (51.1)	1,165,768 (51.3)	1,165,202 (51.2)	1,164,691 (51.1)	4,658,263 (51.2)	0.003
HS attainment,^b^ mean (SD)	0.754 (0.0249)	0.875 (0.131)	0.928 (0.095)	0.965 (0.066)	0.880 (0.221)	0.959
Income ($),^b^ mean (SD)	45,535 (29,418)	65,457 (30,489)	84,809 (37,250)	128,930 (76,887)	81,844 (78,127)	1.067
ECI, mean (SD)	2.66 (4.40)	2.66 (4.49)	2.66 (4.58)	2.68 (4.78)	2.66 (4.56)	0.005
Asthma, *n* (%)	339,023 (14.9)	333,961 (14.7)	333,779 (14.7)	339,364 (14.9)	1,346,127 (14.8)	0.006
CVD, *n* (%)	380,149 (16.7)	381,878 (16.8)	383,127 (16.8)	381,297 (16.7)	1,526,451 (16.8)	0.003
COPD, *n* (%)	98,800 (4.3)	99,631 (4.4)	99,388 (4.4)	99,493 (4.4)	397,312 (4.4)	0.002
Diabetes, *n* (%)	700,324 (30.8)	699,079 (30.7)	698,564 (30.7)	710,586 (31.2)	2,808,553 (30.9)	0.010
Kidney disease, *n* (%)	136,968 (6.0)	136,114 (6.0)	136,214 (6.0)	139,145 (6.1)	548,441 (6.0)	0.005
MSK, *n* (%)	1,203,599 (52.9)	1,208,972 (53.2)	1,209,309 (53.2)	1,199,805 (52.7)	4,821,685 (53.0)	0.010
East North Central, *n* (%)	392,556 (17.3)	392,165 (17.2)	392,145 (17.2)	394,230 (17.3)	1,571,098 (17.3)	0.002
East South Central, *n* (%)	203,272 (8.9)	203,161 (8.9)	203,306 (8.9)	204,558 (9.0)	814,296 (8.9)	0.001
Middle Atlantic, *n* (%)	230,461 (10.1)	229,951 (10.1)	229,646 (10.1)	230,103 (10.1)	920,161 (10.1)	0.001
Mountain, *n* (%)	118,689 (5.2)	118,763 (5.2)	119,065 (5.2)	118,408 (5.2)	474,924 (5.2)	0.002
New England, *n* (%)	152,314 (6.7)	152,384 (6.7)	152,294 (6.7)	152,820 (6.7)	609,811 (6.7)	0.000
Pacific, *n* (%)	459,705 (20.2)	460,973 (20.3)	461,146 (20.3)	461,026 (20.2)	1,842,849 (20.3)	0.001
South Atlantic, *n* (%)	502,969 (22.1)	502,681 (22.1)	502,815 (22.1)	502,182 (22.0)	2,010,646 (22.1)	0.002
West North Central, *n* (%)	108,341 (4.8)	108,373 (4.8)	108,363 (4.8)	109,004 (4.8)	434,082 (4.8)	0.001
West South Central, *n* (%)	105,216 (4.6)	105,291 (4.6)	105,315 (4.6)	105,173 (4.6)	420,995 (4.6)	0.000

^a^ The largest standardized difference between any two quartiles.

^b^ Average proportion of individuals who have attained at least a high school diploma and average household income, at the Census Block Group level. These characteristics were not included in the weighting process.

COPD, chronic obstructive pulmonary disease; CVD, cardiovascular disease; ECI, Elixhauser comorbidity index; HS, high school; MSK, musculoskeletal condition; SD, standard deviation; SES, socioeconomic status.

### TELEHEALTH USE

**Fig. 1.**
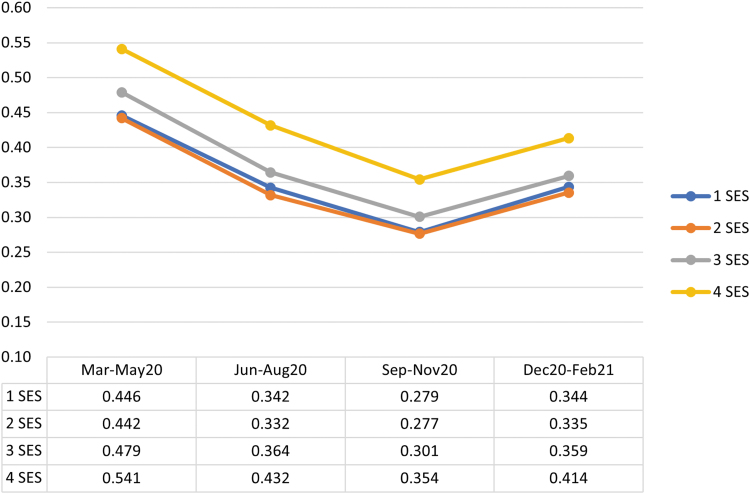
Average number of telehealth visits per member during each 3-month period, by quartile of socioeconomic status (SES). 1–4 represent each quartile, where 1 is the lowest SES.

**Fig. 2.**
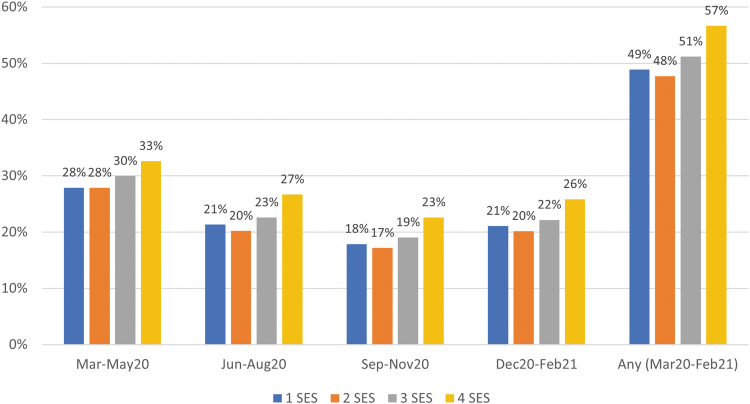
Percentage of individuals with at least one telehealth visit during each 3-month period and during the whole 12-month period, by quartile of SES.

**Fig. 3.**
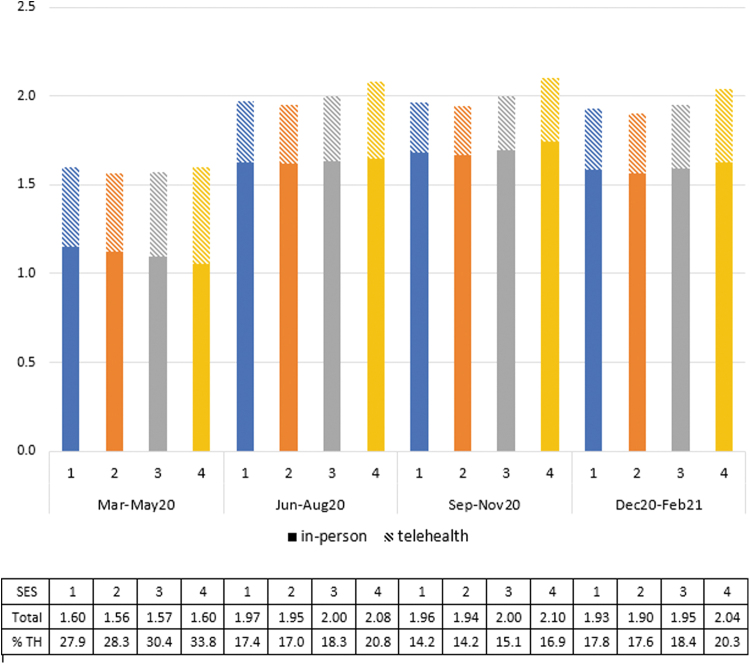
Average number of in-person and telehealth outpatient evaluation and management visits (summing to total) per member during each 3-month period. TH, telehealth; the row “% TH” in the table represents the percentage of total visits that were telehealth.

Across all quartiles of SES, telehealth was most commonly used early in the pandemic (March–May 2020) (*Figs. 1 and 2*), representing 27.9–33.8% (depending on the SES quartile) of all outpatient E&M visits during that time (*Fig. 3*). Total outpatient E&M visits were lowest in March–May 2020, ranging from an average of 1.56–1.60 (depending on the SES quartile) visits per person over the 3-month period, compared with 1.90–2.10 during other quarters. Telehealth usage decreased to its lowest levels in September–November 2020 and then increased in December 2020–February 2021, during the large wave of COVID infections. Unweighted telehealth visits by SES quartile are provided in *Supplementary Appendix SA3*. Between one-tenth and one-fifth of telehealth visits (depending on the SES quartile and timeframe) were coded as audio only, and the percentage of audio-only visits decreased over the course of the year (*Supplementary Appendix SA4*).

### DIFFERENCE BY SES

Telehealth use was higher in Quartile 4 than other quartiles throughout the year, both in terms of average number of visits (*Fig. 1*) and percentage of individuals with a visit (*Fig. 2*). On average, telehealth users in higher SES neighborhoods had slightly more telehealth visits than telehealth users in lower SES neighborhoods (*Supplementary Appendix SA5*). Quartile 4 had 13–18% more telehealth visits (depending on the quarter) than Quartile 3, which had the second highest telehealth usage. Quartile 4 also had a higher percentage of total visits that were telehealth (3.4–5.9 percentage points higher than the other quartiles in March–May 2020, and 1.8–3.7 percentage points higher the rest of the year) (*Fig. 3*).

Although differences in telehealth between Quartiles 1, 2, and 3 were statistically significant (*p* < 0.001 for all pairwise comparisons, except between Quartiles 1 and 2 in March–May 2020 [*p* = 0.146] and September–November 2020 [*p* = 0.360]), these differences were small, with a maximum mean difference of 0.037 visits over each 3-month period. The higher use of telehealth in Quartile 4, as well as more in-person visits September 2020–February 2021 in Quartile 4, resulted in overall higher levels of total outpatient E&M use in Quartile 4 throughout the year, particularly after May 2020. Quartile 2 had slightly lower rates of total E&M visits than other quartiles throughout the year.

Despite their higher usage of telehealth overall, individuals in Quartile 4 had lower use of audio-only visits, accounting for 14.8% of their telehealth visits in March–May 2020 and 10.3% in December 2020–February 2021 (*Supplementary Appendix SA4*). For individuals in Quartile 1, these percentages were 19.3% and 15.2%, respectively.

A more granular view of SES category using deciles instead of quartiles shows a similar pattern (*Supplementary Appendix SA6*). Of note, the third and fourth decile of SES, rather than the first and second, had the lowest telehealth usage. Telehealth usage then gradually increased with each decile after the fourth, with a sharp increase in the highest decile.

## Discussion

Our study found that among a commercially insured population with chronic conditions, individuals living in the highest SES neighborhoods (and particularly the highest decile) stood out as having higher use of telehealth, resulting in greater use of E&M visits overall. The lowest quartile SES group had similar telehealth use to the second quartile and was only slightly lower than the third.

Similar to other studies,^1,4,5^ we found more telehealth usage in individuals living in higher compared with lower income/SES neighborhoods. However, unlike our study, where the *highest* SES neighborhoods were driving this difference, other studies found that the *lowest* median household income quartile^5^ or individuals living in neighborhoods with median household incomes lower than $50,000^1,4^ appeared to have substantially less telehealth usage compared with the general population.

One potential reason that Quartile 4 had more different telehealth use from the other quartiles is that there may be a bigger difference in SES and income in this quartile compared with Quartile 3 than between the other three quartiles; the average median household income by census block group in Quartile 4 was more than $40,000 higher than Quartile 3, whereas there was approximately a $20,000 difference between other quartiles (*Table 1*). It is also possible that this commercial insurer's waiver of copays for telehealth during the pandemic may have further encouraged telehealth rather than in-person visits among low-income populations, thereby reducing disparities in telehealth usage between the lowest and middle SES quartiles. As the observed higher telehealth use in the first and second compared with the third and fourth SES deciles was unexpected, additional analysis exploring the reason for this pattern is warranted.

A concern regarding telehealth use in low-SES populations is that reduced access to internet or video-capable devices,^3^ and lower availability of video telehealth by providers in low-SES neighborhoods,^7^ may result in increased phone-based rather than video-based telehealth. In some cases audio-only visits may be of lower quality than video visits, particularly for conditions where seeing the patient is important for diagnosis (e.g., dermatological conditions). Prior research found that patients who were older than 65 years, Black, Spanish-speaking, and from areas with low broadband access were more likely to use audio-only visits.^10^ Although we found slightly higher rates of coded audio-only visits in the lower compared with higher SES quartiles, rates of audio-only visits were relatively low even among the lowest SES quartile, accounting for less than one fifth of telehealth visits.

### LIMITATIONS

A limitation of this study is that it only includes a commercially insured population, which on average is of higher SES and less vulnerable than the American population generally, and particularly when compared with the Medicaid and uninsured populations. It may be that individuals who were uninsured or who had Medicaid insurance had lower access to telehealth during the first year of the pandemic. In addition, some telehealth visits may have actually been audio only but were not classified with an audio-only–specific CPT code, potentially underestimating the percentage of telehealth visits that were audio only. Finally, SES was measured at the neighborhood (census block) level and may not always be accurate at the individual level.

## Conclusions

Our results indicate that although commercially insured individuals in the highest SES neighborhoods had more utilization of telehealth and outpatient visits overall, those in lower SES neighborhoods did not experience meaningfully lower access to care than those in middle SES neighborhoods during the first year of the COVID-19 pandemic. Although this is encouraging from the standpoint of ensuring access to telehealth and care in general for vulnerable populations, it is important to continue to monitor access to health care services to prevent the growing of health inequities by SES, particularly as cost sharing is reintroduced for telehealth for many health plan members. Further research assessing telehealth and health care usage among Medicaid members and the uninsured is warranted.

## Supplementary Material

Supplemental data

Supplemental data

Supplemental data

Supplemental data

Supplemental data

Supplemental data

## Supplementary Material

Supplementary Appendix SA1

Supplementary Appendix SA2

Supplementary Appendix SA3

Supplementary Appendix SA4

Supplementary Appendix SA5

Supplementary Appendix SA6
