# Supplementary material for: Telehealth and Outpatient Visits Among Individuals with Chronic Conditions by Socioeconomic Status in the First Year of the COVID-19 Pandemic: Observational Cohort Study
Source: Telemed J E Health. 2023 Jul 4;29(7):1105–10. doi: 10.1089/tmj.2022.0233 (PMC10354307; doi:10.1089/tmj.2022.0233)
Supplement: Supplemental data [file Supp_AppendixSA1.docx]

**Appendix 1. Distribution of patient paid cost per telehealth visit, in dollars**

|  | **Mean** | **25th Percentile** | **Median** | **75th Percentile** | **99th Percentile** |
| --- | --- | --- | --- | --- | --- |
| **SES Quartile 1** |  |  |  |  |  |
| Mar-May 20 | 6.75 | 0 | 0 | 0 | 100.00 |
| Jun-Aug 20 | 8.19 | 0 | 0 | 7.32 | 103.28 |
| Sep-Nov 20 | 10.96 | 0 | 0 | 15.05 | 108.97 |
| Dec20-Feb21 | 19.14 | 0 | 2.34 | 25.00 | 153.84 |
| **SES Quartile 2** |  |  |  |  |  |
| Mar-May 20 | 7.15 | 0 | 0 | 0 | 105.54 |
| Jun-Aug 20 | 8.43 | 0 | 0 | 7.82 | 108.00 |
| Sep-Nov 20 | 11.07 | 0 | 0 | 15.95 | 109.08 |
| Dec20-Feb21 | 20.30 | 0 | 4.81 | 25.00 | 164.01 |
| **SES Quartile 3** |  |  |  |  |  |
| Mar-May 20 | 7.38 | 0 | 0 | 0 | 109.12 |
| Jun-Aug 20 | 8.53 | 0 | 0 | 7.04 | 112.75 |
| Sep-Nov 20 | 11.22 | 0 | 0 | 15.00 | 119.62 |
| Dec20-Feb21 | 21.12 | 0 | 2.50 | 25.00 | 176.68 |
| **SES Quartile 4** |  |  |  |  |  |
| Mar-May 20 | 8.17 | 0 | 0 | 0 | 123.46 |
| Jun-Aug 20 | 8.88 | 0 | 0 | 5.00 | 124.75 |
| Sep-Nov 20 | 11.82 | 0 | 0 | 15.00 | 131.23 |
| Dec20-Feb21 | 23.17 | 0 | 3.61 | 29.50 | 196.76 |

SES = socioeconomic status
