# Supplementary material for: Telehealth and Outpatient Visits Among Individuals with Chronic Conditions by Socioeconomic Status in the First Year of the COVID-19 Pandemic: Observational Cohort Study
Source: Telemed J E Health. 2023 Jul 4;29(7):1105–10. doi: 10.1089/tmj.2022.0233 (PMC10354307; doi:10.1089/tmj.2022.0233)
Supplement: Supplemental data [file Supp_AppendixSA2.docx]

**Appendix 2. Unweighted baseline characteristics by quartile of socioeconomic status**

| SES Quartile | 1 | 2 | 3 | 4 |
| --- | --- | --- | --- | --- |
| N | 573,156 | 573,052 | 573,253 | 573,163 |
| *Mean (SD)* |  |  |  |  |
| Age | 51.18 (16.11) | 51.16 (16.54) | 51.05 (17.15) | 50.69 (18.11) |
| Elixhauser comorbidity index | 2.86 (2.29) | 2.72 (2.29) | 2.63 (2.27) | 2.40 (2.22) |
| HS attainment ^a^ | 0.755 (0.122) | 0.878 (0.063) | 0.928 (0.047) | 0.963 (.035) |
| Income ($)^a^ | 45,505 (14,521) | 65,115 (14,873) | 84,873 (18,580) | 131,944 (40,198) |
| *n (%)* |  |  |  |  |
| Female | 297,531 (51.9%) | 293,283 (51.2%) | 292,183 (51.0%) | 290,762 (50.7%) |
| Asthma | 77,758 (13.6%) | 79,038 (13.8%) | 86,824 (15.1%) | 93,384 (16.3%) |
| CVD | 96,059 (16.8%) | 95,241 (16.6%) | 95,959 (16.7%) | 98,240 (17.1%) |
| COPD | 10,133 (5.3%) | 28,771 (5.0%) | 24,053 (4.2%) | 17,239 (3.0%) |
| Diabetes | 207,137 (36.1%) | 187,202 (32.7%) | 169,713 (29.6%) | 138,566 (24.2%) |
| Kidney disease | 37,834 (6.6%) | 34,993 (6.1%) | 33,626 (5.9%) | 30,166 (5.3%) |
| MSK | 289,471 (50.5%) | 302,335 (52.8%) | 307,983 (53.7%) | 320,805 (56.0%) |
| East North Central | 91,191 (15.9%) | 128,596 (22.4%) | 110,859 (19.3%) | 65,576 (11.4%) |
| East South Central | 89,340 (15.6%) | 58,264 (10.2%) | 35,898 (6.3%) | 22,085 (3.9%) |
| Middle Atlantic | 29,669 (5.2%) | 43,586 (7.6%) | 65,421 (11.4%) | 92,695 (16.2%) |
| Mountain | 22,423 (3.9%) | 29,647 (5.2%) | 35,743 (6.2%) | 31,616 (5.5%) |
| New England | 18,139 (3.2%) | 31,136 (5.4%) | 52,632 (9.2%) | 55,836 (9.7%) |
| Pacific | 111,430 (19.4%) | 82,005 (14.3%) | 101,925 (17.8%) | 166,671 (29.1%) |
| South Atlantic | 153,951 (26.9%) | 138,150 (24.1%) | 117,172 (20.4%) | 95,896 (16.7%) |
| West North Central | 23,374 (4.1%) | 36,119 (6.3%) | 31,171 (5.4%) | 18,435 (3.2%) |
| West South Central | 33,593 (5.9%) | 25,542 (4.5%) | 22,429 (3.9%) | 24,351 (4.2%) |

^a^ Average proportion of individuals who have attained at least a high school diploma and average household income, at the Census Block Group level.

CVD=cardiovascular disease; COPD=chronic obstructive pulmonary disease; HS=high school; MSK=musculoskeletal condition; SD=standard deviation; SES=socioeconomic status
