# Supplementary material for: Telehealth and Outpatient Visits Among Individuals with Chronic Conditions by Socioeconomic Status in the First Year of the COVID-19 Pandemic: Observational Cohort Study
Source: Telemed J E Health. 2023 Jul 4;29(7):1105–10. doi: 10.1089/tmj.2022.0233 (PMC10354307; doi:10.1089/tmj.2022.0233)
Supplement: Supplemental data [file Supp_AppendixSA3.docx]

**Appendix 3. Unweighted average number of telehealth visits by quartile of socioeconomic status**

**Caption:** Unweighted average number of telehealth visits per member during each 3-month period. SES=socioeconomic status; 1-4 represent each quartile, where 1 is the lowest SES.
