# Supplementary material for: Telehealth and Outpatient Visits Among Individuals with Chronic Conditions by Socioeconomic Status in the First Year of the COVID-19 Pandemic: Observational Cohort Study
Source: Telemed J E Health. 2023 Jul 4;29(7):1105–10. doi: 10.1089/tmj.2022.0233 (PMC10354307; doi:10.1089/tmj.2022.0233)
Supplement: Supplemental data [file Supp_AppendixSA4.docx]

**New: Appendix 4. Average number of telehealth visits by quartile of socioeconomic status, split into audio-only visits versus other (video or unspecified) visits**

**Caption:** Average number of telehealth visits per member during each 3-month period. 1-4 represent each socioeconomic quartile, where 1 is the lowest. Percentages listed below the timeframe indicate the percentage of telehealth visits that were coded as audio-only.
